# Ultrasound features of medullary thyroid cancer as predictors of biological behavior

**DOI:** 10.1186/s40644-021-00402-w

**Published:** 2021-04-09

**Authors:** Jingzhu Zhao, Xiangqian Zheng, Ming Gao, Sheng Zhang, Xinwei Yun, Jiadong Chi, Guangwei Xu

**Affiliations:** 1 Departments of Thyroid and Neck Tumor, Tianjin Medical University Cancer Institute and Hospital, National Clinical Research Center for Cancer, Key Laboratory of Cancer Prevention and Therapy, Tianjin, Tianjin’s Clinical Research Center for Cancer, Tianjin, 300060 P. R. China; 2 Department of Diagnostic and Therapeutic Ultrasonography, Tianjin Medical University Cancer Institute and Hospital, National Clinical Research Center for Cancer, Key Laboratory of Cancer Prevention and Therapy, Tianjin, Tianjin’s Clinical Research Center for Cancer, Tianjin, 300060 P. R. China

**Keywords:** Medullary thyroid cancer, TI-RADS, Ultrasound, Serum Ct, Recurrence

## Abstract

**Background:**

Medullary thyroid cancer (MTC) has more aggressive behavior and poor prognosis. Ultrasound (US) has facilitated the qualitative diagnosis of thyroid nodules, however, some MTC may be diagnosed as a benign nodule on ultrasound because ultrasound features of malignancy are lacking. The aim of the study was to investigate the association between ultrasound features and biological behavior of MTC.

**Methods:**

Ultrasound findings and medical records of patients with MTC between Jan 2015 to Jun 2017 were retrospectively reviewed at Tianjin Medical University Cancer Institute and Hospital. MTC were categorized using modified TI-RADS classification, then were classified as “malignant” (m-MTC) or “US-low-suspicious” (l-MTC). We compared the biological behavior between the two groups, and further analyzed the risk factors for the recurrence.

**Results:**

A total of 78 patients were enrolled, of which 55 m-MTC (70.5%) and 23 l-MTC (29.5%) were identified. The N staging of the m-MTC was significantly higher than that of l-MTC(*P* = 0.000). The preoperative serum Ct level in m-MTC were significantly higher than that of l-MTC(*P* = 0.035). Biochemical cure were more frequent in l-MTC than that of m-MTC (*P* = 0.002). Disease recurrence rates were 19.7% (14 of 71). Disease recurrence was more frequent in m-MTC than that of l-MTC (*P* = 0.013). Disease recurrence was positively associated with extrathyroid extension (*P* = 0.047), N staging (*P* = 0.003), preoperative serum Ct level (*P* = 0.009) and negatively associated with biochemical cure(*P* = 0.000). In multivariable Cox regression analysis, extrathyroid extension and biochemical cure were independent risk factors for recurrence of MTC.

**Conclusions:**

L-MTC has a more indolent character than m-MTC. The extrathyroid extension and biochemical cure were independent risk factors for recurrence of MTC.

## Background

Medullary thyroid cancer (MTC) originates from thyroid C cells and accounts for about 5% of thyroid malignancy [1]. MTC has more aggressive behavior than differentiated thyroid cancers and up to 13.4% of thyroid cancer-related deaths due to MTC [2, 3]. Therefore, early diagnosis of MTC is important for improving long-term prognosis.

Ultrasound is the crucial tool for the preliminary evaluation of thyroid nodules [4, 5]. Several classification systems have been proposed in order to standardize the assessment of thyroid nodules using ultrasonography [6, 7]. Most classification systems, including TI-RADS, are primarily applicable to papillary thyroid carcinoma. There was only a few studies researched the application of TI-RADS in assessing MTC [8, 9], and the applicability of TI-RADS in MTC patients is still not sufficient.

Therefore, the aim of our study was to more fully investigate the ultrasound features of MTC and to analyze the association ultrasound features with its biological behavior.

## Materials and methods

### Patients

There were 86 patients who had been operated for MTC over the period from Jan 2015 to Jun 2017 at Tianjin Medical University Cancer Institute and Hospital. The preoperative diagnosis primarily based on high serum calcitonin levels and preoperative neck ultrasonography. 8 patients were excluded as they had undergone thyroid excision in other hospital. Finally, 78 patients were enrolled in the study. This study was approved by the ethics committee of our hospital, and informed consent was obtained from all patients.

### Sonographic image analysis according to modified TI-RADS

Ultrasound examinations were performed with a 5–12 MHz linear array transducer (iU22; Philips Diagnostic Ultrasound System, Bothell, WA). Ultrasound images of each thyroid nodule were recorded in our institutional database. Retrospective review of ultrasound images was performed by two experienced reviewers. All reviewers were blinded to the clinical pathology results and categorized the nodules according to modified TI-RADS [9, 10].If the diagnosis were discordant, the third reviewer served to achieve consensus. In brief, nodules were categorized on ultrasound by composition, echogenicity, presence of calcification, margin, shape and vascularization according to the standard methodology from previously published reports [9, 10]. The categories were as follows: TI-RADS 1: normal thyroid gland, no nodule, no need to follow. TI-RADS 2: benign (risk = 0%), nodule without any of the six high suspicious ultrasound features, suggested follow-up every year. TI-RADS 3 (risk≤5%): probably benign, nodule with any one of the six high suspicious ultrasound features, suggested follow-up every 6 months or FNAB if necessary. TI-RADS 4: suspicious malignant, 4a: nodule with any two or three of the six high suspicious ultrasound features, (risk range, 6 to 45%); 4b: nodule with any four of the six high suspicious ultrasound features, (risk range, 46 to 75%); 4c: nodule with any five or six of the six high suspicious ultrasound features, (risk range, 76 to 95%), suggested FNAB or surgery. TI-RADS 5: certainly malignant (risk > 95%), one or more suspect lymph node(s) were associated with a thyroid nodule, suggested surgery. TI-RADS 6: malignant (risk = 100%), nodule confirmed by cytology or pathology.

According to their ultrasound features, all nodules were categorized as “malignant” (m-MTC) or “US-low-suspicious” (l-MTC). The m-MTC were included as TI-RADS 4b,4c,5,6, and the l-MTC were included as TI-RADS 2,3,4a.

### Serum calcitonin levels

All patients had values for serum calcitonin measured using chemiluminescent immumoassay (reference range, < 5 pg/mL for women, < 8.4 pg/mL for men). The preoperative and postoperative calcitonin level were recorded. The level of serum calcitonin is greater than 50–100 pg/mL, a diagnosis of MTC is common [11]. A biochemical cure was defined by a calcitonin level within the reference range.

### Statistical analysis

All statistical analyses were performed using SPSS software (SPSS, Chicago, IL, USA). The clinicopathologic features of all patients were compared statistically between l-MTC and m-MTC. The Student’s t-test was used for the continuous numerical variables. The Chi-square test was used for categorical variables. The multivariate logistic regression analysis to determine independent risk factors of recurrence. Time to recurrence was calculated from the date of first surgery until recurrence. The last follow-up time is Feb 10, 2020. A value of *P* < 0.05 was considered statistically significant.

## Results

The present study consisted of 78 patients (38 males and 40 females) with histologically proved MTC, and their mean age was 50.9 ± 11.8 years (range, 14–75 years). 76 patients underwent thyroidectomy with lymph node dissection for detecting MTC, and the other two patients, misdiagnosed as benign nodule, underwent thyroidectomy without lymph node dissection. Preoperative serum Ct was available in all 78 cases, and postoperative serum Ct was available in 63 cases.

### Comparison of clinicopathologic features of patients with l-MTC or m-MTC

According to the ultrasound criteria for risk evaluation, 23 out of 78 cases were classified as l-MTC [TI-RADS 2 (*n* = 2, 2.6%);TI-RADS 3 (*n* = 3, 3.8%);TI-RADS 4a (*n* = 18, 23.1%)] and 55 out of 78 cases as m-MTC[(TI-RADS 4b (*n* = 16, 20.5%); TI-RADS 4c (*n* = 7, 9.0%); TI-RADS 5 (*n* = 32, 41.0%)]. The comparison of the clinicopathologic features of patients with l-MTC and m-MTC was shown in Table 1. The proportion of female in l-MTC was higher than in m-MTC (*P* = 0.010). The mean rank of preoperative Ct/tumor size in m-MTC is larger than that in l-MTC(*P* = 0.042). Cervical lymph node metastasis (central lymph node metastasis *P* = 0.000, lateral lymph node metastasis *P* = 0.004 and N staging *P* = 0.000) were more frequent in m-MTC than that of l-MTC. The preoperative serum Ct level in m-MTC is higher than that in l-MTC (*P* = 0.035). The proportion of biochemical cure in l-MTC was significantly higher than in m-MTC(*P* = 0.002). The other variables, such as age, tumor size, multifocality, extrathyroid extension, T staging and M staging did not show a significant difference between the two groups.

**Table 1:** Comparison of clinical characteristics of patients with l-MTC and m-MTC

Variable	N	l-MTC (*n* = 23)	m-MTC (*n* = 55)	*P*
Age (years)		47.0 ± 14.8	52.6 ± 10.0	0.114^a^
Preoperative Ct/tumor size (pg/mL/mm)		31.43	42.87	0.042^b^
Gender				0.010
Female	40	17 (73.9)	23 (41.8)	
Male	38	6 (26.1)	32 (58.2)	
Tumor size (cm)		1.70 ± 1.52	1.75 ± 1.21	0.910
≤ 1	30	12 (52.2)	18 (32.7)	0.107
>1	48	11 (47.8)	37 (67.3)	
Multifocality				0.843
Yes	25	7 (30.4)	18 (32.7)	
No	53	16 (69.6)	37 (67.3)	
Extrathyroid extension				0.892
Yes	45	13 (56.5)	32 (58.2)	
No	33	10 (43.5)	23 (41.8)	
Central lymph node metastasis^c^				0.000
Yes	35	2 (9.5)	33 (60.0)	
No	41	19 (90.5)	22 (40.0)	
Lateral lymph node metastasis^d^				0.004
Yes	30	1 (20.0)	29 (12.1)	
No	8	4 (80.0)	4 (87.9)	
Preoperative Ct level (pg/mL)				0.035
≤ 100	19	10 (43.5)	9 (16.4)	
100–1000	31	6 (26.1)	25 (45.4)	
> 1000	28	7 (30.4)	21 (38.2)	
Biochemical cure^e^				0.002
Yes	39	18 (90.0)	21 (48.8)	
No	24	2 (10.0)	22 (51.2)	
T staging				0.892
T1 + T2	33	10 (43.5)	23 (41.8)	
T3 + T4	45	13 (56.5)	32 (58.2)	
M staging				1.000
M1	4	0 (0.0)	4 (7.3)	
M0	74	23 (100.0)	51 (92.7)	
N staging				0.000
N0	35	20 (87.0)	15 (27.3)	
N1a	13	2 (8.7)	11 (20.0)	
N1b	30	1 (4.3)	29 (52.7)	

^a^t test; ^b^ Mann-Whitney U test; ^c^ 2 patients underwent thyroidectomy without lymph node dissection; ^d^ Only 38 patients were performed lateral lymph node dissection; ^e^ Postoperative serum Ct is available in 63 cases

### The univariate and multivariate analysis of recurrence

4 patients had underwented palliative surgery, and 3 patients were detected distant metastasis when initial surgery. So 7 patients were not included in the recurrence analysis. Median follow-up time is 35(1–69) months. In the overall analysis of MTC, disease recurrence rates were 19.7% (14 of 71). The univariate analysis of recurrence was shown in Table 2.

**Table 2:** The univariate analysis for recurrence in MTC patients

Variable	N	Recurrence		*P*
		No	Yes	
Age (years)		49.5 ± 12.1	53.4 ± 9.3	0.266^a^
Preoperative Ct/tumor size (pg/mL/mm)		32.15	51.68	0.002^b^
Gender				0.768
Female	38	31 (54.4)	7 (50.0)	
Male	33	26 (45.6)	7 (50.0)	
Tumor size (cm)		1.48 ± 0.96	1.81 ± 1.11	0.271
≤ 1	30	26 (45.6)	4 (28.6)	0.247
>1	41	31 (54.4)	10 (71.4)	
Multifocality				0.917
Yes	22	17 (29.8)	5 (35.7)	
No	49	40 (70.2)	9 (64.3)	
Extrathyroid extension				0.047
Yes	39	28 (49.1)	11 (78.6)	
No	32	29 (50.9)	3 (21.4)	
Central node metastasis^c^				0.000
Yes	30	18 (32.7)	12 (85.7)	
No	39	37 (67.3)	2 (14.3)	
Lateral node metastasis^d^				1.000
Yes	26	18 (78.3)	8 (72.7)	
No	8	5 (21.7)	3 (27.3)	
T stage				0.166
T1 + T2	32	28 (49.1)	4 (28.6)	
T3 + T4	39	29 (50.9)	10 (71.4)	
N stage				0.003
N0	33	32 (56.1)	1 (7.1)	
N1a	12	7 (12.3)	5 (35.7)	
N1b	26	18 (31.6)	8 (57.1)	
Preoperative Ct level (pg/mL)				0.009
≤ 100	16	16 (28.1)	0 (0.0)	
100–1000	31	25 (43.9)	6 (42.9)	
> 1000	24	16 (28.1)	8 (57.1)	
Biochemical cure^e^				0.000
Yes	39	37 (78.7)	2 (15.4)	
No	21	10 (21.3)	11 (84.6)	
Classification of TI-RADS				0.013
l-MTC	22	22 (38.6)	0 (0.0)	
m-MTC	49	35 (61.4)	14 (100.0)	

^a^t test; ^b^ Mann-Whitney U test; ^c^ 2 patients underwent thyroidectomy without lymph node dissection; ^d^ Only 38 patients were performed lateral lymph node dissection; ^e^ Postoperative serum Ct is available in 63 cases,and 3 of these patients had underwented palliative surgery

**Fig. 1:**
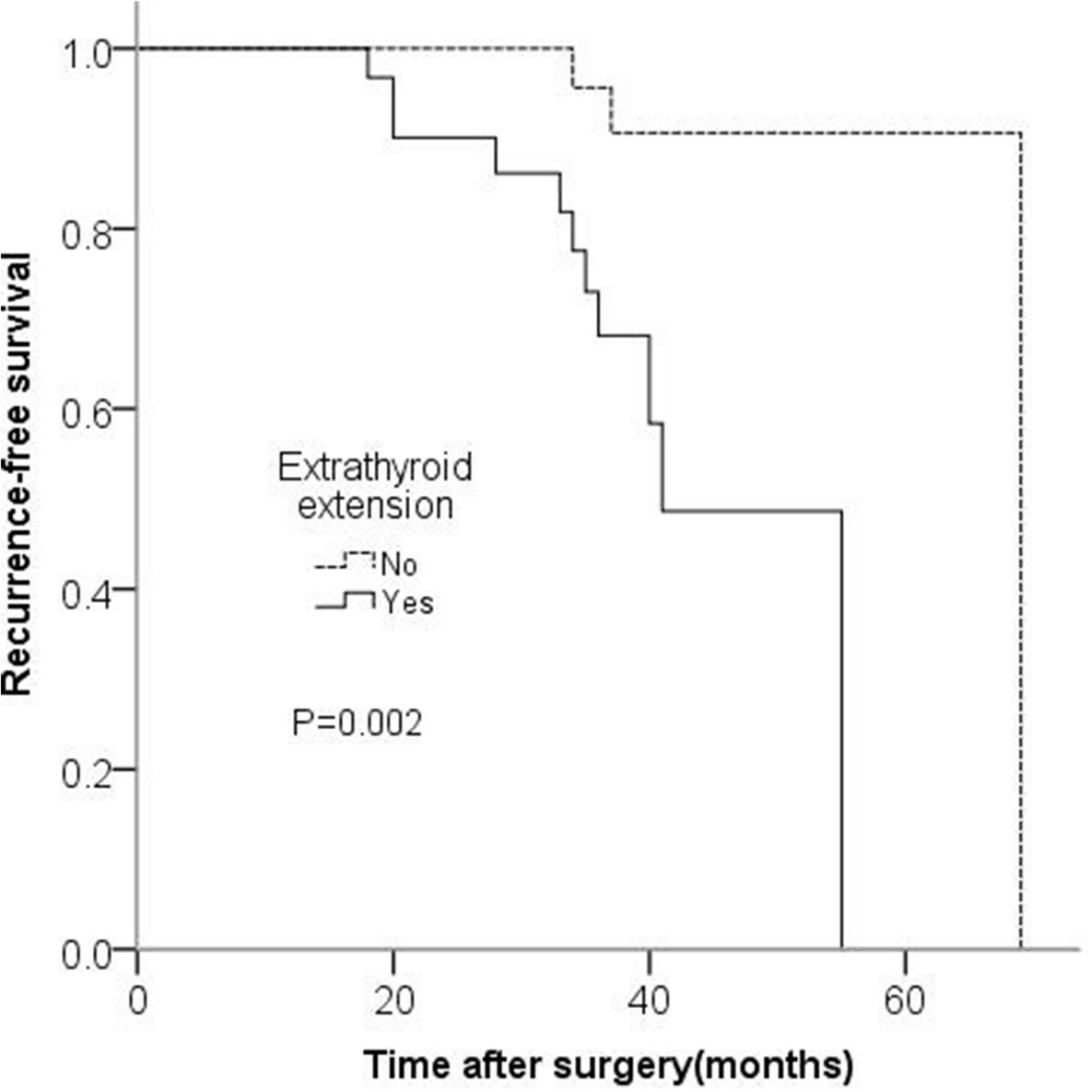
Kaplan-Meier analysis of recurrence-free survival. The mean recurrence-free time of patients with extrathyroid extension was obviously shorter than that of patients without extrathyroid extension (P_log-rank_ = 0.002)

**Fig. 2:**
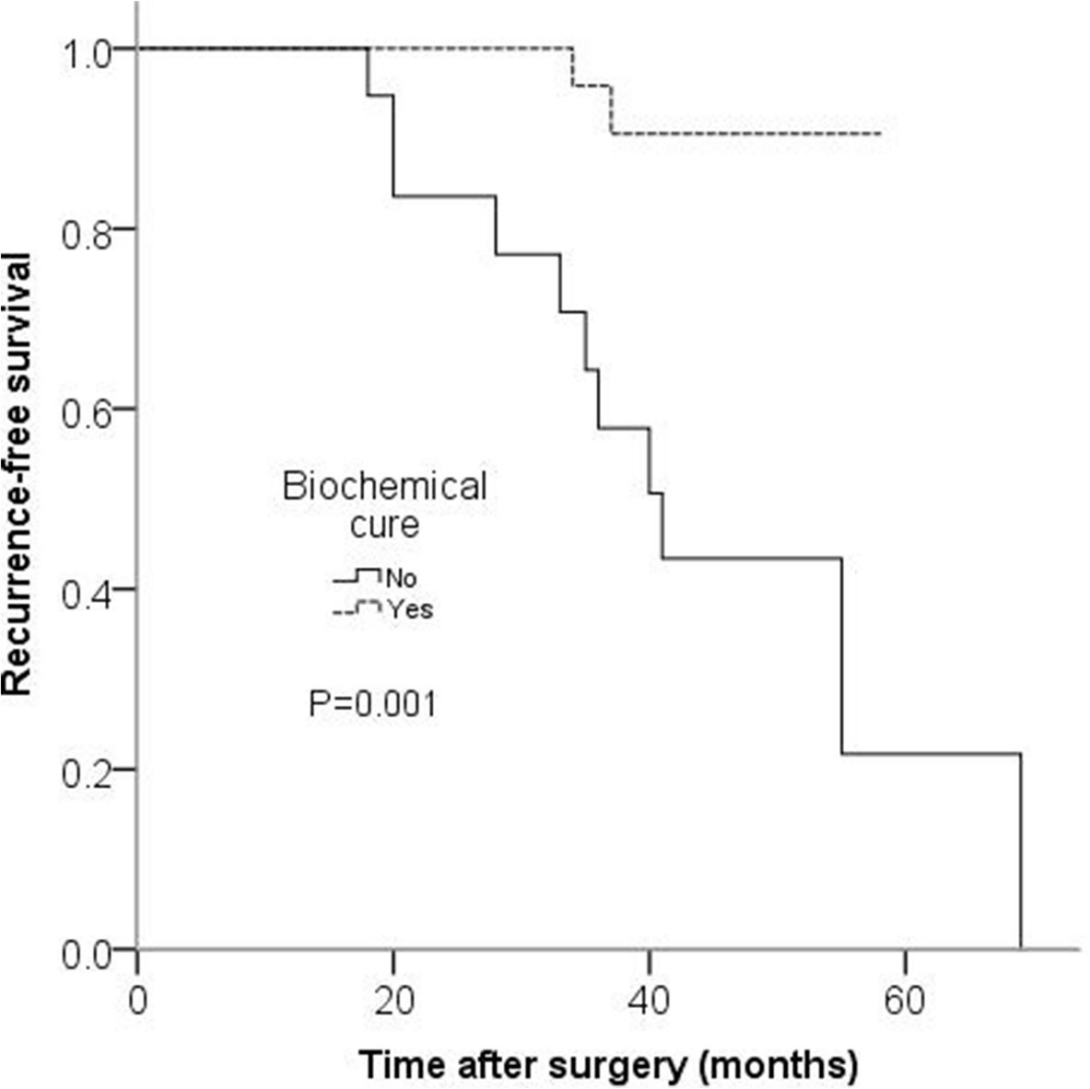
Kaplan-Meier analysis of recurrence-free survival. The mean recurrence-free time of patients with biochemical cure was obviously longer than that of patients without biochemical cure (P_log-rank_ = 0.001)

Disease recurrence was negatively associated with biochemical cure(*P* = 0.000), and positively associated with extrathyroid extension (*P* = 0.047), central node metastasis (*P* = 0.000),N staging(*P* = 0.003) and preoperative serum Ct level(*P* = 0.009). Disease recurrence was more frequent in m-MTC than that of l-MTC (*P* = 0.013). There was no association in recurrence with gender, age, tumor size, multifocality and T staging. Furthermore, in multivariable Cox regression analysis, extrathyroid extension and biochemical cure were independent risk factors for recurrence of MTC (Table 3). Kaplan-Meier analysis showed that the mean recurrence-free time of patients with extrathyroid extension was obviously shorter than that of patients without extrathyroid extension(*P* = 0.002), and the mean recurrence-free time of patients with biochemical cure was obviously longer than that of patients without biochemical cure(*P* = 0.001) (Figs. 1 and 2).

**Table 3:** The multivariate analysis of clinicopathological features for recurrence in MTC patients

Variable	B	S.E.	Wald	df	Sig.	Exp(B)	95% C.I. for Exp(B)	
							Lower	Upper
Extrathyroid extension	1.653	0.791	4.366	1	0.037	5.223	1.108	24.624
Biochemical cure	−1.913	0.785	5.947	1	0.015	0.148	0.032	0.687

### Challenge of diagnosis in l-MTC patients

**Fig. 3:**
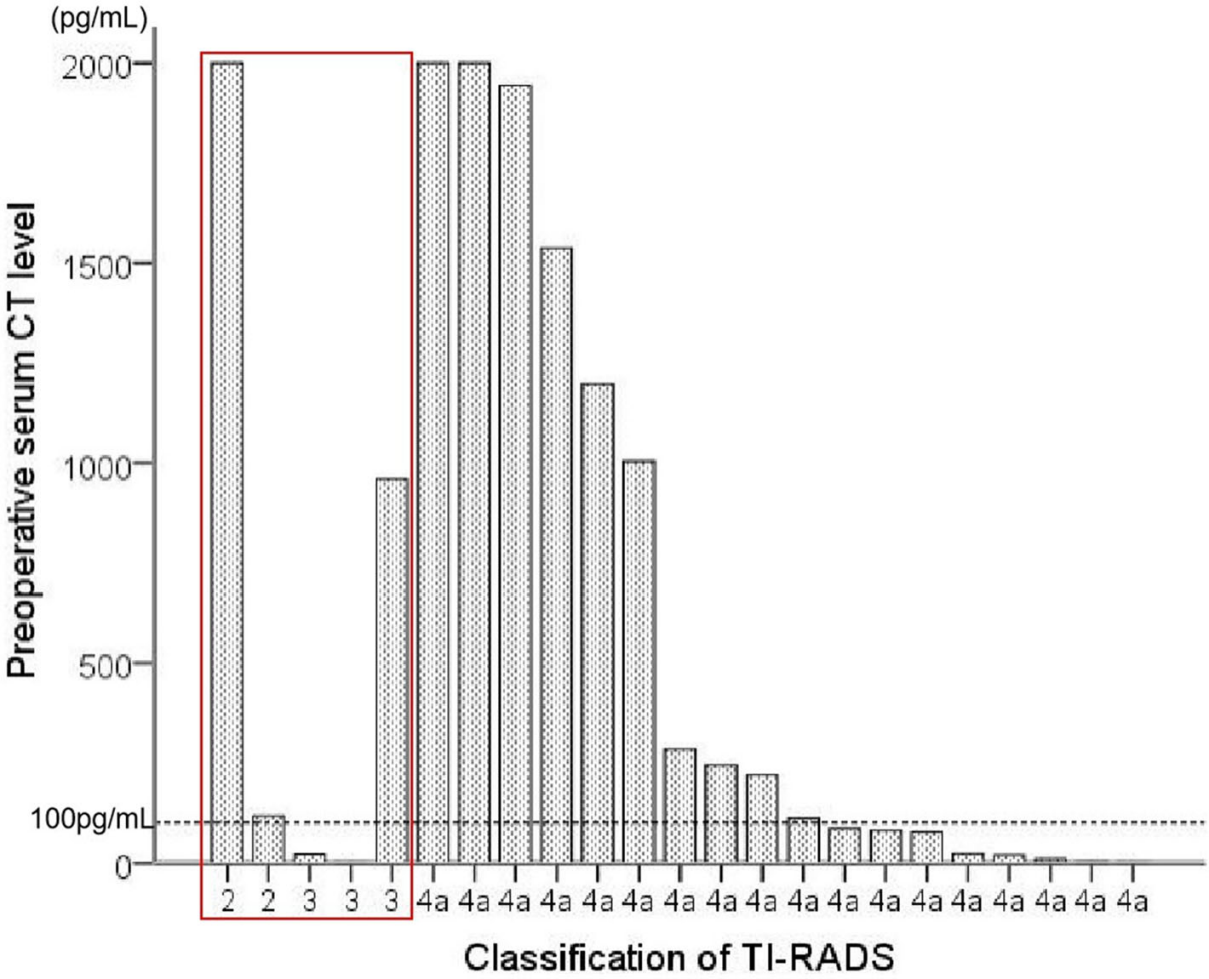
The preoperation calcitonin level of 23 patients with l-MTC. 5 patients were categorized as TI-RADS 2 and 3 (in the red box)

**Fig. 4:**
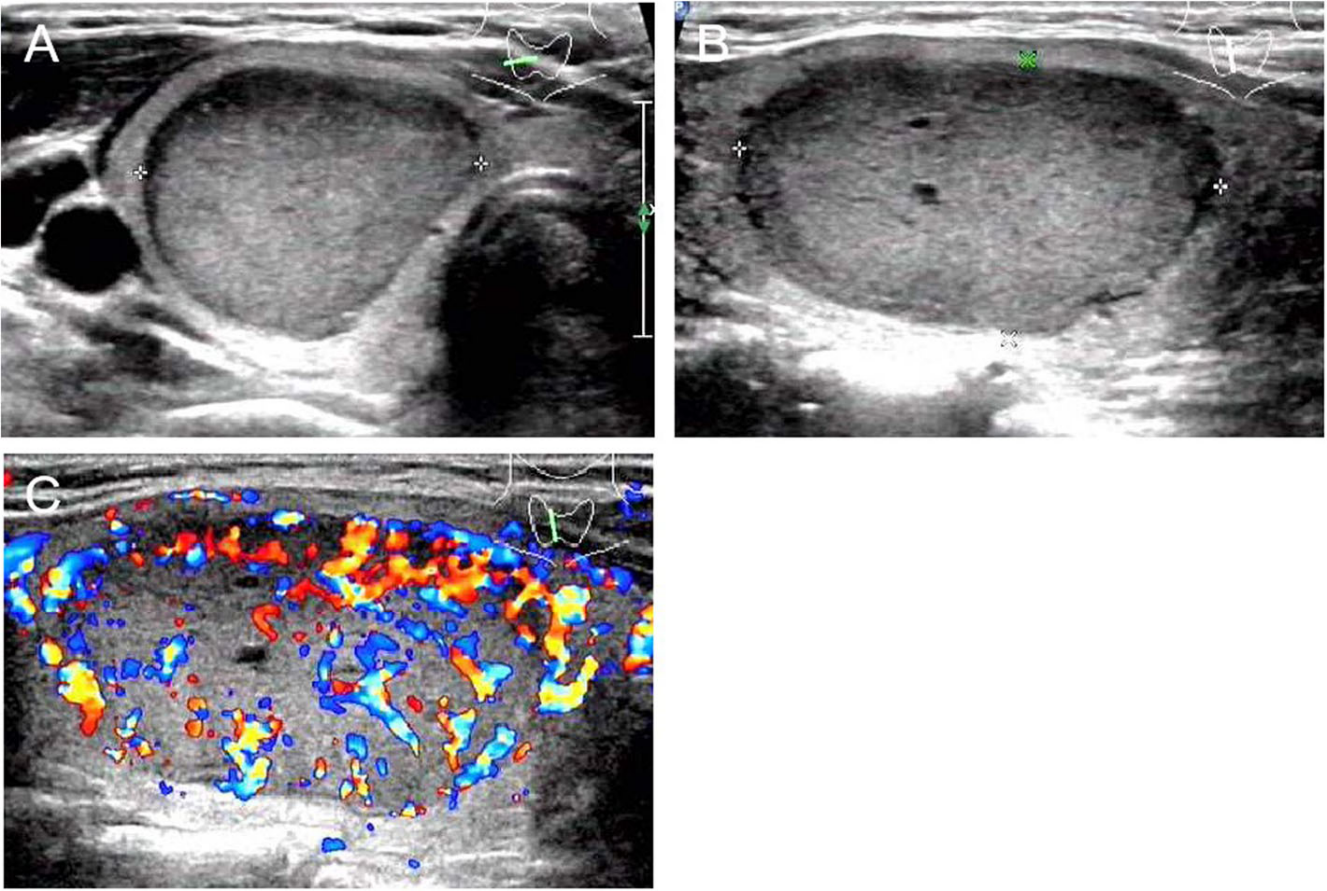
US images of medullary thyroid carcinoma nodules categorized by TI-RADS 2. Female patient, 32 years old, 3.8 cm × 2.7 cm × 2.2 cm. The serum calcitonin level was > 2000 pg/mL. **a** and **b** Lesion was solid, iso- to hypoechoic echogenicity, well-defined, ovoid shape, small anechoic zone. **a** A/T < 1. **c** Enhanced blood flow

**Fig. 5:**
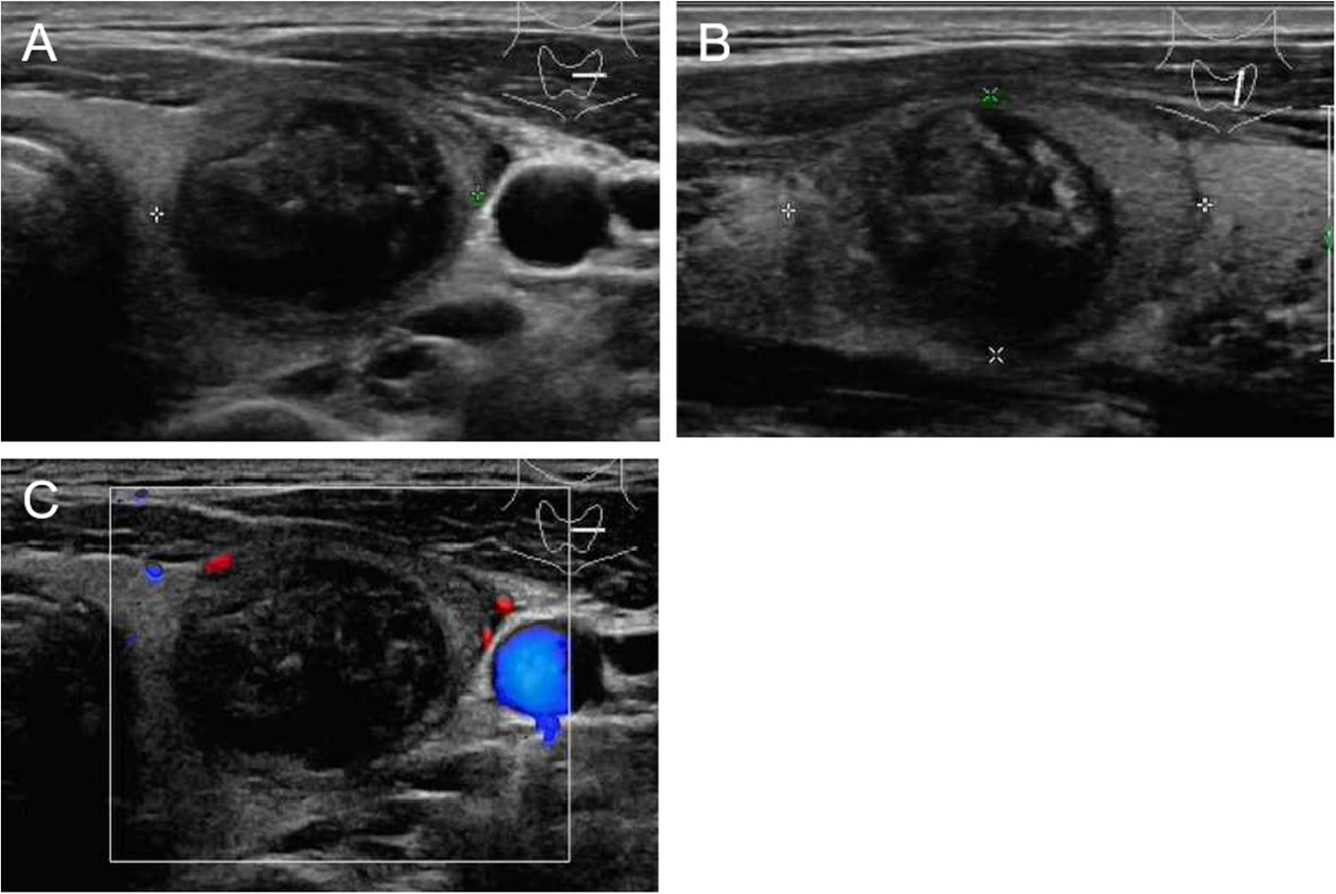
US images of medullary thyroid carcinoma nodules categorized by TI-RADS 3. Male patient, 40 years old, 3.06 cm × 2.36 cm × 1.85 cm. The serum calcitonin level was <2 pg/mL. **a** and **b** Lesion was solid, hypoechoic, ill-defined, ovoid to round in shape, microcalcifications. **a** A/T < 1. **c** Absent blood flow

**Fig. 6:**
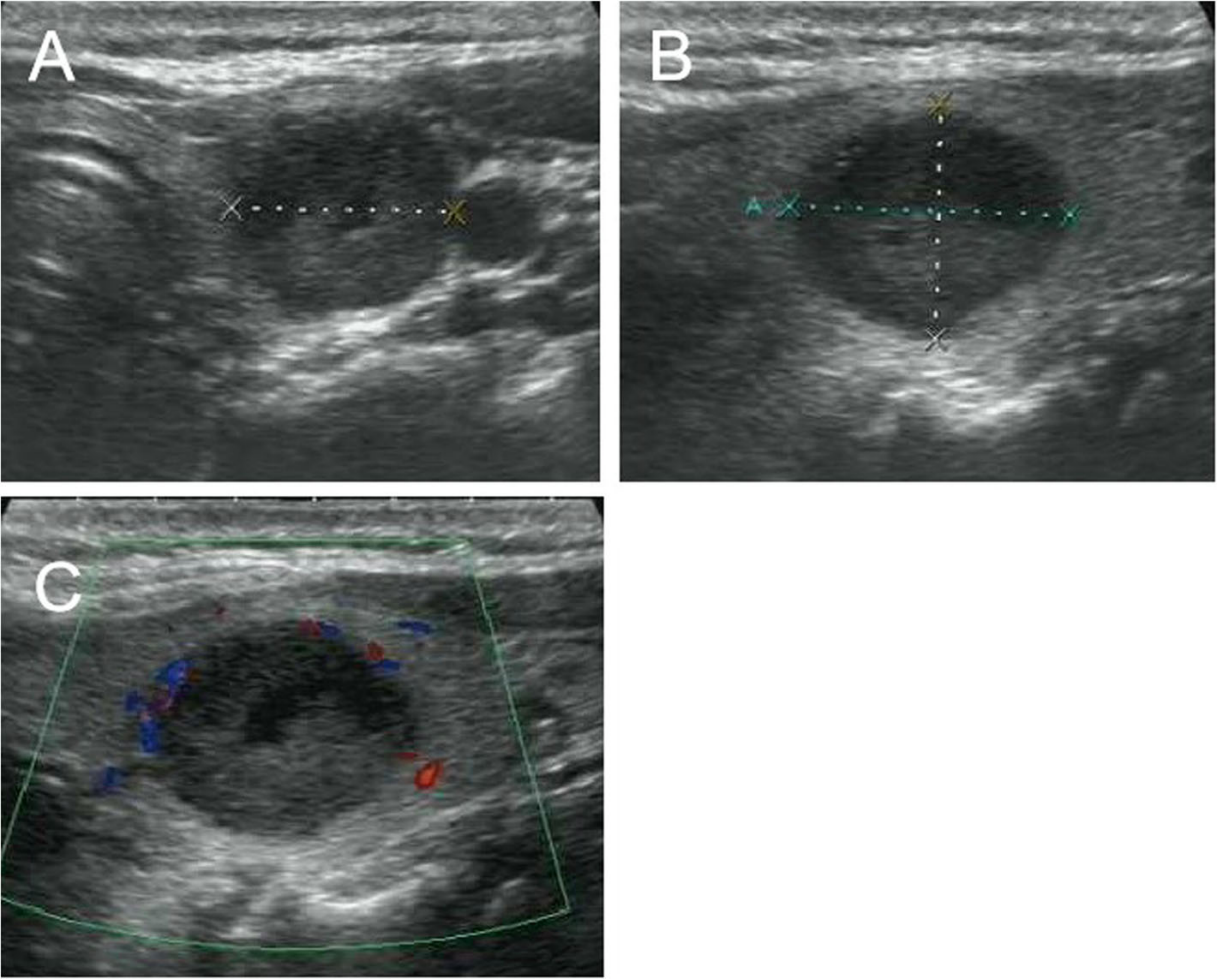
US images of medullary thyroid carcinoma nodules categorized by TI-RADS 3. Female patient, 48 years old, 1.8 cm × 1.4 cm × 1.4 cm. The serum calcitonin level was 960 pg/mL. **a** and **b** Lesion was solid with a little of fluid sonolucent area, hypoechoic, well-defined, ovoid to round in shape. **a** A/T < 1. **c** Absent blood flow

In the present study, 5 patients, who were categorized as TI-RADS 2 and 3, were eventually diagnosed with medullary thyroid cancer by histopathology (Fig. 3, in the red box). Immunohistochemical examination were performed in 5 patients, including Cal, TG, TTF1, Syn, Ki-67 and CK-pan. The preoperative serum Ct level of the 5 patients were 117 pg/mL, > 2000 pg/mL, 20.9 pg/mL, 960 pg/mL and<2 pg/mL, respectively (Table 4). Ultrasonic images of thyroid nodules were showed (Figs. 4, 5 and 6).

**Table 4:** A summary of clinicopathological features in 5 patients

N	Graded by TI-RADS	Serum Ct level (pg/mL)	Immunostaining					
			Cal	TG	TTF1	Syn	Ki-67	CK-pan
1	2	117	+	–	+	NA	2–3%	+
2	2	> 2000	+	–	+	NA	<1%	+
3	3	20.9	+	–	+	+	NA	+
4	3	960	+	–	+	+	<1%	+
5	3	<2	+	–	+	–	NA	–

*NA* not available

## Discussion

The present results showed that TI-RADS effectively stratified the risk of malignancy in the group of MTC, providing a basis for further treatment. The biological characteristics of m-MTC differed from those of l-MTC. Lymph node metastasis were significantly more frequent in m-MTC than that of l-MTC. The level of serum calcitonin was significantly higher in m-MTC than that of l-MTC. Furthermore, the proportion of biochemical cure in l-MTC was significantly higher than in m-MTC.

Our study indicated that there were no obvious difference of mean diameter between l-MTC and m-MTC. However, the previous study reported that l-MTC were significantly larger than m-MTC [12, 13]. It may is because that l-MTC may be diagnosed at a later stage than m-MTC because of their benign ultrasonographic features in published literature, and l-MTC might be diagnosed at early stage in our hospital because of routine measurement of serum Ct in evaluating thyroid nodular.

In the previous studies, approximately one-third of MTC patients had benign ultrasonic characteristics [12–15], which mainly include solid, hypoechogenicity, ovoid to round in shape and smooth margin. The results of our study showed that based on the modified TI-RADS, 70.5%(55/78) of MTC could be categorized as “high-risk” by ultrasonic examination, while a nonnegligible portion (29.5%) was low risk or no risk. Because l-MTC accounts for a considerable proportion of all MTC patients, a full understanding of the benign ultrasonic characteristics of MTC is vital to detect and diagnosis the lesion in earlier stage. However, some thyroid nodules indeed distinguish difficultly from benign nodule, and the underlying mechanism of formation needs further study.

Several studies have emphasized that early diagnosis of MTC is very important for surgical cure and improving prognosis [16, 17]. The previous studies found that suspicious ultrasonic characteristics were less frequence in MTC compared with PTC [4],which might lead to delayed diagnosis and treatment. Furthermore, as the previous study [12–15], about 29.5% of MTC were classified as l-MTC in our study. So, measurement of serum Ct is a another useful diagnostic tool for MTC. However, routine measurement of serum calcitonin remains controversial [4, 18–20], due to cost-effective problem and the low prevalence of the disease. As a clinician, I think that the costs of detecting one MTC patient are quite reasonable by routine measurement of Ct, compared to the potential costs of missing the diagnosis of this treatable malignancy, even prognosis or life. In our hospital, routine measurement of serum Ct is recommended by most clinician. In fact, routine measurement of serum Ct indeed plays an important role in detecting MTC in earlier stage in clinical practice. Therefore, routine measurement of serum calcitonin should be recommended in initial evaluation thyroid nodules.

Our study showed that disease recurrence was positively associated with N staging and negatively associated with serum Ct level and biochemical cure. Due to misdiagnosed as benign nodule, two patients who only underwent thyroidectomy without lymph node dissection did not show recurrence. None of l-MTC patients relapsed, while 14 patients with m-MTC showed recurrence. As the previous study [13], disease recurrence was more frequent in m-MTC than that of l-MTC. In all, l-MTC has a more indolent biological behavior than m-MTC. Regarding disease recurrence, extrathyroid extension and biochemical cure were independent risk factors for recurrence of MTC.

The present study has several limitations. Firstly, in view of the retrospective study, there might be inherent selection bias. Secondly, our study was performed at a single center and included a small patient population. A multicenter prospective study may be necessary. Finally, there were no genetic information in all tumor, which may lead to different growth patterns.

## Conclusion

In conclusion, modified TI-RADS could predict the biological behavior of MTC. A comprehensive understanding of the ultrasonic characteristics for l-MTC is important in cases of a suspicious MTC without malignant ultrasonic characteristics. L-MTC has a more indolent character than m-MTC.The extrathyroid extension and biochemical cure were independent risk factors for recurrence of MTC.

## Data Availability

The datasets supporting the conclusion of this article available from the corresponding author on reasonable request.

## Abbreviations

TI-RADS: Thyroid Imaging Reporting and Data System
MTC: Medullary thyroid carcinoma
Ct: Calcitonin
